# The impact of a mixed exercise intervention on the physical and mental health of left-behind children: a randomized controlled trial combining sports games and ball sports

**DOI:** 10.3389/fpsyg.2026.1829202

**Published:** 2026-07-20

**Authors:** Tao Fang, Anjie Wang, Yan Wang, Han Li

**Affiliations:** 1 College of Physical Education, Anhui Normal University, Wuhu, China; 2 School of Physical Education, Anhui Polytechnic University, Wuhu, China

**Keywords:** ball sports, left-behind children, mental health, physical education, physical fitness, randomized controlled trial, school-based intervention, sports games

## Abstract

**Background:**

Left-behind children (LBC) in rural China often have limited opportunities for regular physical activity and may face heightened psychological difficulties. School physical education (PE) provides a practical setting in which structured interventions can be delivered to this group. This study examined whether a 12-week PE program combining cooperative sports games with ball-sport activities was related to changes in physical fitness and psychological health among rural LBC.

**Methods:**

Forty sixth-grade LBC from a rural boarding primary school were randomly allocated to a mixed training group (MTG, *n* = 20) or a control group (CONG, *n* = 20). The MTG completed the mixed exercise program during regular PE classes for 12 weeks, with three 90-min sessions each week. The CONG continued with routine PE. Psychological health was measured using the Mental Health Test (MHT). Physical fitness was assessed with standard school-based indicators, including vital capacity, 50-m dash, 8 × 50-m shuttle run, rope skipping, sit-ups, sit-and-reach, and body mass index (BMI). Group-by-time effects were examined using repeated-measures ANOVA.

**Results:**

Following the 12-week period, the MTG showed greater improvements in selected psychological and physical outcomes compared with the CONG. Significant group-by-time interactions were found for MHT total score (*p* < 0.001, *d* = −1.07), interpersonal anxiety (*p* = 0.007, *d* = −0.73), and self-blame tendency (*p* = 0.002, *d* = −1.41). Among physical fitness outcomes, significant group-by-time interactions were observed for vital capacity (*p* < 0.001, *d* = 0.97), 8 × 50-m shuttle run performance (*p* < 0.001, *d* = −0.90), 50-m dash performance (*p* = 0.002, *d* = −0.78), and rope skipping (*p* = 0.024, *d* = 0.73). However, BMI and sit-ups showed no significant group-by-time interactions. No exercise-related injuries or other adverse events occurred during the study.

**Conclusion:**

A 12-week mixed exercise program embedded in routine PE was associated with selective improvements in psychological health and physical fitness among rural LBC. These findings provide preliminary support for the potential value of combining cooperative sports games with ball-sport activities in school PE. Larger multi-site studies with longer follow-up are needed to confirm these findings.

## 1 Introduction

China’s rapid urbanization and economic change have led many rural workers to move to cities for employment and higher income (Sun et al., 2024). This migration has reshaped rural family arrangements and contributed to the growth of left-behind children (LBC) in rural areas (Chen, 2024). LBC are children who remain in rural communities while one or both parents work away from home and who are cared for by grandparents or other relatives (Zhou et al., 2023). Most LBC live in rural areas of China, where caregiving and daily living conditions differ from those of non-left-behind children. Such living and caregiving conditions may increase developmental risks in both physical and mental health (Wang et al., 2025).

Physical and mental health in childhood are important for later development (Subramanyam et al., 2024). Studies show that LBC face more mental health problems than their peers, including depression (Ruan et al., 2025), self-harm (Zheng et al., 2025), and antisocial behavior (Qi et al., 2025). These problems often appear together with social withdrawal and difficulties in social adjustment (Hung et al., 2025). Over time, these conditions can affect physical health and the ability to adapt to daily life (Fauk et al., 2025). These risks point to the need for school-accessible interventions that support emotional wellbeing and create more opportunities for peer interaction among LBC.

Physical activity is known to benefit children’s physical and mental health, including cardiorespiratory fitness, motor competence, emotional wellbeing, and psychosocial adjustment (Biddle and Asare, 2011; Janssen and Leblanc, 2010). In recent years, several types of exercise-based programs have been used with LBC. These include artistic sports activities (Hu et al., 2024; Li et al., 2025; Zhou et al., 2023), targeted physical training programs (Li and Ren, 2024), and game-based sports activities (Liu et al., 2025). These studies suggest that physical activity can support both physical fitness and mental wellbeing in this group. However, existing interventions often emphasize either emotional engagement through playful or expressive activities, or physical improvement through more structured exercise training. Fewer studies have integrated peer interaction, progressive exercise load, and motor skill development within the same school-based physical education framework.

The health challenges faced by LBC do not result from a single physical or psychological cause. They are linked to limited family companionship (Zhuang et al., 2025), less varied daily activities (Chen and Zhang, 2025), and fewer chances for stable peer interaction (Zhang et al., 2025), among other factors (Xiong et al., 2024). This perspective is also supported by evidence from social-contextual research, which indicates that children’s physical activity and health behaviors are jointly influenced by individual, social, and environmental factors (Sallis et al., 2000). Physical and mental difficulties often occur together, and social interaction remains limited. From the perspective of Self-Determination Theory, physical education activities that foster autonomy, competence, and relatedness may support adaptive motivation and psychological functioning (Ryan and Deci, 2000). A social connectedness perspective further suggests that stable interpersonal bonds and a perceived sense of belonging are important for psychological adjustment (Baumeister andLeary, 1995). For LBC, school-based physical education may be particularly suitable when it combines structured physical activities with repeated opportunities for cooperative peer interaction. Many existing exercise programs primarily facilitate emotional regulation through games or enhance physical fitness through skills training. However, these approaches do not always provide adequate exercise load and meaningful social interaction at the same time. Single-focus interventions may therefore be less suited to the combined physical and psychosocial needs of LBC; by contrast, a model that links cooperative play with physical training may better support both physical fitness and psychological health.

Sports games are generally accessible to children and can be used in a range of activity settings (Bowman and Chang, 2023). These activities may foster peer interaction and emotional engagement within group settings, particularly when they are organized in participatory and low-competition formats (Aksović et al., 2023; Weiss et al., 1996). Sports games have been linked to reduced negative affective experiences (Moya-Higueras et al., 2023), increased peer interaction (Guo et al., 2024), and resilience-related psychological resources (Cheng and Ebrahimi, 2023). This may be particularly beneficial for LBC experiencing adverse living conditions. However, game-based activities alone may not provide sufficient stimulus for physiological fitness components or structured motor skill development (Nagorna et al., 2024). Ball sports place higher demands on movement skills and physical fitness and help improve endurance, speed, and coordination (Lesmawan et al., 2025; Nancheva and Ivanov, 2024). They also require cooperation and communication between teammates, which supports social interaction (Haoran et al., 2023; Wang et al., 2024). However, some children may experience pressure when ball-sport tasks are too difficult or overly competitive. Combining cooperative sports games with ball sports may therefore provide a more balanced intervention structure by linking social participation, movement skill learning, and physical conditioning within regular school physical education.

Against this background, the present study used a randomized controlled trial design to examine a mixed exercise program combining cooperative sports games with ball sports. The main contribution of this study lies in embedding this mixed model within routine physical education classes for rural LBC, rather than adding an extracurricular training program or relying on a single activity form. This design treats routine PE as a setting with modifiable elements, including activity structure, content organization, participation format, and exercise load, that can be adjusted to better support both physical and psychological health. By integrating cooperative game-based activities, structured ball-sport training, and progressive exercise-load control, the study examined whether restructuring regular PE lessons was associated with changes in psychological health and physical fitness. The primary outcomes were the MHT total score, vital capacity, and the 8 × 50-m shuttle run. Secondary outcomes included MHT subscales and other physical fitness indicators, including 50-m dash, rope skipping, sit-ups, sit-and-reach, and BMI. It was hypothesized that, compared with children receiving routine physical education, children in the mixed training group would show greater reductions in psychological difficulties and greater improvements in selected physical fitness indicators, particularly cardiorespiratory function, endurance, speed, and coordination. Given the relatively short intervention duration and the non-strength-specific nature of the program, changes in BMI and strength-endurance indicators were expected to be limited.

## 2 Materials and methods

### 2.1 Participants

**FIGURE 1:**
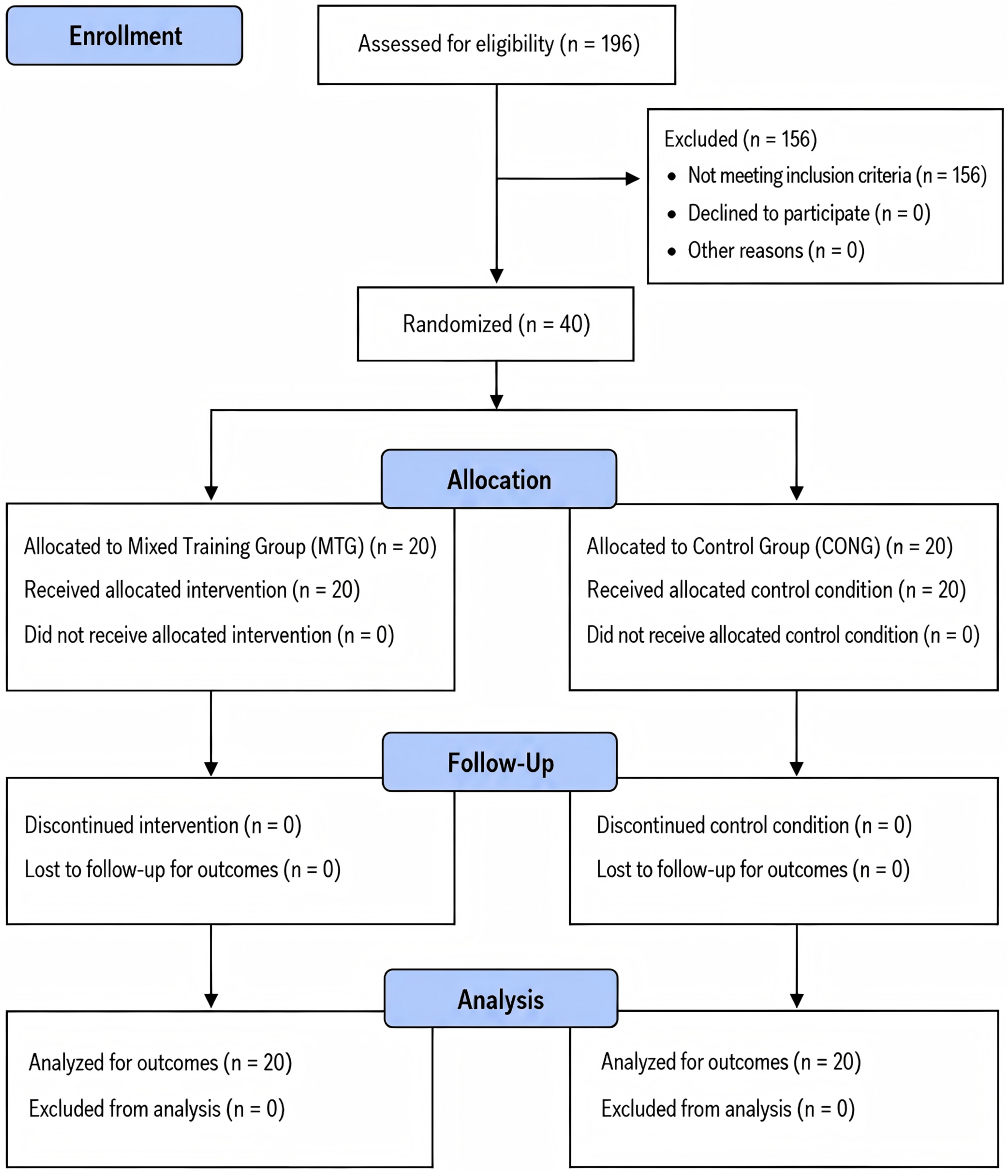
CONSORT flow diagram of participant enrollment, allocation, follow-up, and analysis.

This study was conducted in a rural primary school in J County, Province A, China. This school is a non-profit boarding school that provides educational services to children including LBC, orphans, children from single-parent families, and children with disabilities. Students had similar conditions in learning, daily life, and physical activity. In this study, LBC were operationally defined as children who remained in rural communities while one or both parents had migrated for work for more than 6 months and who were cared for by grandparents or other relatives. Although orphans and children from single-parent families were also present in the school and may experience varying degrees of psychosocial vulnerability and health-related difficulties, they were not included in the target population of this study because their family structures and sources of parental absence differed from the operational definition of LBC based on parental labor migration and prolonged parent–child separation. Students were screened according to the operational definition of LBC described above. Eligible participants were required to be in the sixth grade and to have experienced separation from one or both migrant parents for at least 6 months. Children were not enrolled if they were orphans, came from single-parent families, or had physical or neurological conditions that could affect safe participation in physical activity. Participation was voluntary. The required sample size was estimated using G*Power software (version 3.1.9.7, Universität Düsseldorf, Germany). The parameters were set at α = 0.05 and statistical power = 0.80 (1 - β). Because no pilot data were available for this mixed exercise intervention among rural LBC, the calculation used Cohen’s conventional moderate effect size for a group × time repeated-measures ANOVA (*f* = 0.25), with an assumed repeated-measures correlation of *r* = 0.50 (Cohen, 2013). Based on this estimate, the minimum sample size required for this study was 34 participants. The recruitment process took place from September 1 to September 25, 2025. This period included screening and enrollment. After screening based on multiple criteria, 40 left-behind children were recruited as participants in this study. They were randomly assigned in a 1:1 ratio to either the mixed training group (MTG, *n* = 20) or the control group (CONG, *n* = 20) (Figure 1).

The random allocation sequence was generated by an independent researcher, who was not involved in the recruitment or intervention process, using a computer-generated random number table. A block randomization procedure with a block size of 4 was employed to ensure balanced group sizes throughout the enrollment process. To ensure allocation concealment, the assignments were kept in sequentially numbered, opaque, sealed envelopes. A second researcher, blinded to the allocation sequence, was responsible for enrolling the participants and opening the envelopes to assign them to their respective groups. Allocation concealment was ensured during group assignment. Due to the nature of the intervention, participants and the physical education teacher delivering the sessions were not blinded to group allocation. However, outcome assessors responsible for physical fitness testing were blinded to group assignment to reduce assessment bias.

Baseline measurements were conducted prior to the intervention to characterize participants in both groups with respect to age, gender, height, weight, body mass index (BMI), Functional Movement Screen (FMS) score, and duration of moderate-to-vigorous physical activity (MVPA). Statistical analyses revealed no significant between-group differences in baseline characteristics (all *p* > 0.05), suggesting baseline comparability between the groups (Table 1).

**TABLE 1:** Baseline characteristics of participants.

Characteristic	MTG (*n* = 20)	CONG (*n* = 20)	*p*
Age, years	12.18 ± 0.33	12.21 ± 0.36	0.790
Male, n (%)	11 (55%)	10 (50%)	0.750
Female, n (%)	9 (45%)	10 (50%)	–
Height, cm	152.6 ± 6.8	151.9 ± 7.1	0.750
Weight, kg	42.9 ± 6.7	41.8 ± 6.2	0.590
BMI, kg/m^2^	18.38 ± 0.90	18.11 ± 0.68	0.279
FMS score	14.55 ± 1.85	14.30 ± 1.90	0.670
MVPA, min/week	185 ± 42	178 ± 45	0.580

Values are presented as mean ± SD unless otherwise indicated. Gender is presented as n (%). The dash indicates that the *p*-value was not repeated; the *p*-value shown in the Male row refers to the overall chi-square test of sex distribution between groups. MTG, mixed training group; CONG, control group; BMI, body mass index; FMS, Functional Movement Screen; MVPA, moderate-to-vigorous physical activity. Other *p*-values were derived from independent-samples *t*-tests.

### 2.2 Intervention design

Consistent with the social-contextual and self-determination perspectives introduced above, the mixed PE intervention was designed to reorganize routine PE lessons into a structured setting that combined physical activity, peer interaction, cooperative participation, and progressive task experiences. The program combined sports games with ball-sport activities and was delivered as part of the regular school PE curriculum. No additional extracurricular training sessions were added. After randomization, the intervention was carried out from October 13, 2025, to January 2, 2026. Public holidays and non-school days were not included in the training schedule. The intervention lasted 12 weeks, and post-intervention assessments were completed by January 9, 2026. The trial followed the planned protocol and was not stopped early. The MTG and CONG had the same class schedule and setting, with three 90-min PE sessions each week, including a 10-min break. Each MTG session included three fixed parts.

(1) Game-based intervention segment (20–25 min)

**FIGURE 2:**
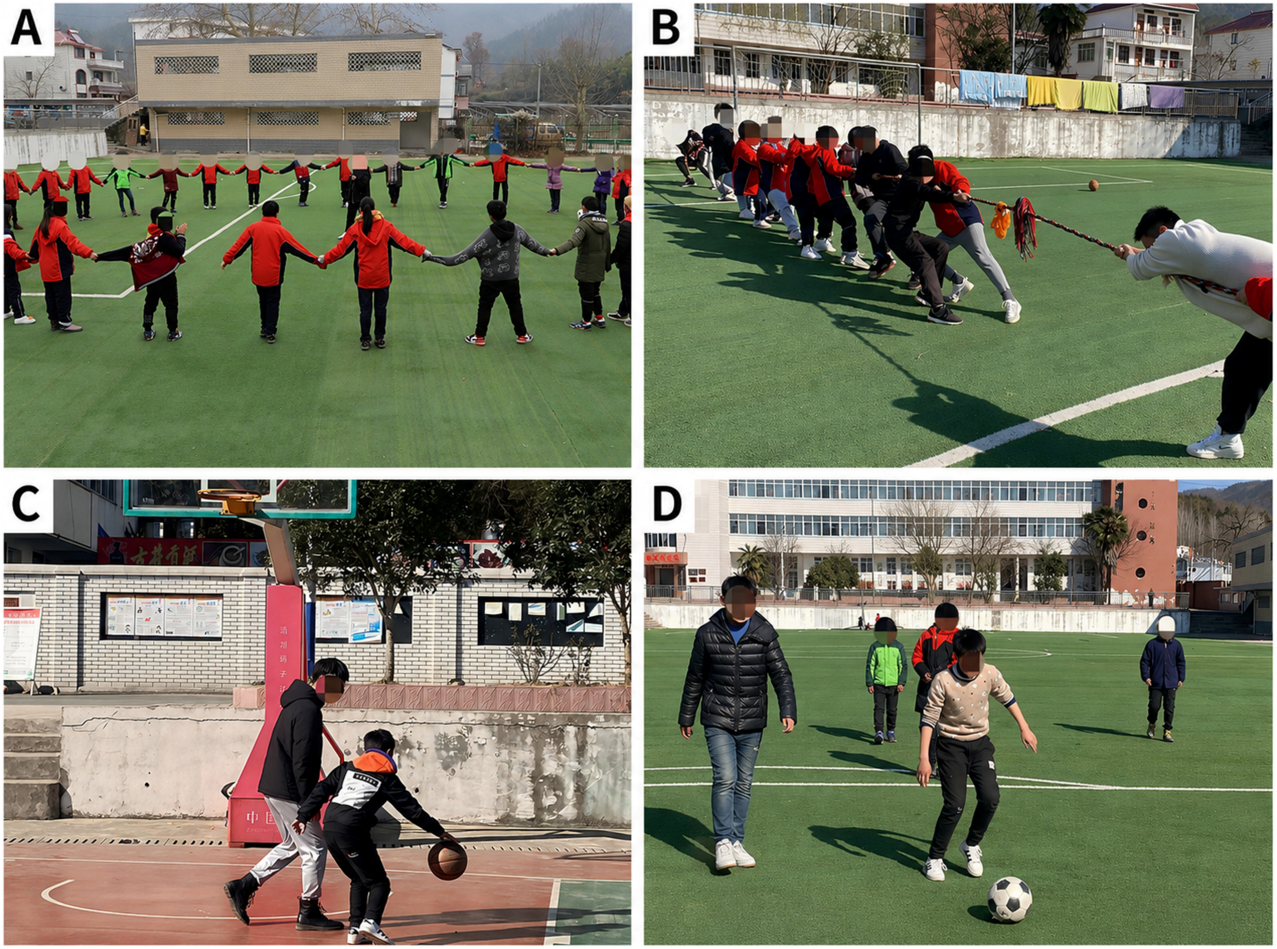
Representative activities included in the mixed exercise intervention. **(A,B)** Representative cooperative physical games used in the game-based intervention segment: **(A)** circle-based cooperative task; **(B)** modified tug-of-war game. **(C,D)** Representative core ball-sport activities used in the ball-sports intervention segment: **(C)** basketball-based activity; **(D)** soccer-based activity.

This part consisted of cooperative physical games commonly used in school PE. The games became progressively more cooperative and demanding across the 12 weeks. In the early stage, students became familiar with group participation and shared rules. Later, the activities required more cooperation, mutual assistance, and collective effort. During weeks 1–4, the games focused on simple group tasks and peer familiarity. Students worked in close group formations and practiced coordinated movements during exercise. Examples included circle-based collaboration (Figure 2A) and reaction-focused games. During weeks 5–8, rule-based chasing and rescue games were introduced, such as freeze-and-rescue tag. These activities required students to help teammates and respond to shared cues during play. During weeks 9–12, the games shifted toward group resistance tasks, including modified tug-of-war games (Figure 2B). All games were organized as low-competition, participation-oriented activities, with more emphasis on peer connection than individual performance. Details of game selection and organization are provided in Table 2, with representative photographic examples shown in Figures 2A,B.

**TABLE 2:** Design framework and representative cooperative physical games used in the game-based intervention segment.

Stage (duration)	Representative game	Core procedures	Intervention rationale (social and psychological target)
Stage 1: participation and foundation building (weeks 1–4)	Circle-based tasks (e.g., circle pulse)	Participants formed a closed circle by holding hands. Following rhythmic instructions, they moved clockwise or counterclockwise, or performed squats together without breaking the circle.	Promoted peer familiarity, physical proximity, and comfort in group movement.
Stage 1: participation and foundation building (weeks 1–4)	Rhythmic synchronization (e.g., turnip squat)	Groups were assigned different labels, such as colors or names. Each group performed a specific movement immediately after being called by another group in a rhythmic sequence.	Encouraged attention to peers, rule following, and a basic sense of group connection.
Stage 2: collaboration and skill consolidation (weeks 5–8)	Dynamic network cooperation (e.g., fishermen’s net)	Two catchers held hands to form a net and tagged other participants. Tagged participants joined the net by holding hands, expanding the group until the remaining participants were captured.	Encouraged coordination, nonverbal communication, and shared movement control.
Stage 2: collaboration and skill consolidation (weeks 5–8)	Social rescue interaction (e.g., freeze-and-rescue tag)	Participants tagged by the chaser froze in place. They could return to the game only after a teammate successfully touched and rescued them.	Promoted mutual assistance, trust, and peer reliance through help-and-rescue actions.
Stage 3: application and comprehensive improvement (weeks 9–12)	Strategic defense and team synergy (e.g., dodgeball/sandbag)	The inner team dodged sandbags thrown by the outer team. Team members could shield vulnerable peers or catch the sandbag to revive eliminated teammates.	Encouraged protective cooperation, team awareness, and collective response under moderate competition.
Stage 3: application and comprehensive improvement (weeks 9–12)	High-intensity collective resistance (e.g., Tug-of-war)	Two teams performed a collective resistance task. Success depended on synchronized effort and coordinated physical output toward a shared goal.	Strengthened team cohesion, synchronized effort, and collective goal pursuit.

The games listed in this table represent common cooperative physical activities used across the intervention stages. Activities were adjusted and rotated between sessions while keeping the same task structure and social focus. The table presents the functional design of the intervention rather than a full list of all activities used. Figures 2A,B provide representative photographic examples of selected cooperative physical games described in this table.

(2) Core ball sports section (55–60 min).

After the game-based activity, students completed a structured ball-sports section. Basketball- and soccer-based activities were alternated across the intervention period (Figures 2C,D). The content became more demanding over time in terms of technical difficulty, coordination requirements, and physical intensity. During weeks 1–4, the focus was on basic motor skills, simple drills, and low-intensity practice. During weeks 5–8, the training moved from individual skill practice to partner and small-group coordination. Typical tasks included ball-control drills, movement exercises, and cooperative practice situations in which students had to adjust their actions in relation to others. During weeks 9–12, small-sided games were used to combine skill execution with simple tactical decision-making and higher levels of physical activity.

(3) Concluding cool-down (3–5 min).

At the end of each session, students completed 3–5 min of cool-down activities, mainly stretching and light movement. These activities were used to support recovery and relaxation. The progression of training content and instructional focus across stages is summarized in Table 3.

**TABLE 3:** Progressive structure of the 12-week mixed exercise intervention combining sports games and ball sports.

Intervention stage	Period	Game-based segment	Ball-sports segment	Progressive focus
Stage 1: participation and foundation building	Weeks 1–4	Cooperative, interactive, and low-competitive games were used to promote participation and familiarity with the rules.	Basic ball-sport movement training and foundational skill practice were conducted in a low-contact setting.	Participation motivation and basic movement patterns.
Stage 2: collaboration and skill consolidation	Weeks 5–8	Games required simple communication and collaboration, with a greater proportion of group cooperative tasks.	Partner and small-group coordination tasks were completed around basic ball skills.	Motor-skill consolidation and peer collaboration.
Stage 3: application and comprehensive improvement	Weeks 9–12	Cooperative gameplay was continued to enhance team interaction and task completion.	Small-sided competitions and scenario drills were conducted using simplified rules.	Task complexity, exercise load, and skill transfer/integration.

Exercise load was planned with attention to the participants’ age and the practical conditions of school PE. In the MTG, intensity was guided by target heart-rate zones rather than maximal exercise testing. Target ranges were calculated with the age-predicted maximal heart rate equation (HRmax ≈ 208 − 0.7 × age) and checked against international recommendations for moderate-to-vigorous physical activity in children (Mahon et al., 2010). Because the participants were approximately 12 years old on average, the estimated maximal heart rate was about 199 beats per minute. The workload increased across three stages (Table 4). The first stage maintained activity at a moderate level. The second stage introduced more moderate-to-vigorous activity. The final stage included higher-intensity tasks, while still keeping the workload within the planned target range. This arrangement allowed the physical stimulus to increase alongside the technical and movement demands of the ball-sport activities.

**TABLE 4:** Progressive exercise intensity design during the 12-week intervention.

Stage	Training focus	Period	Target HR, beats/min	Percentage of HRmax
Basic stage	Skill learning	Weeks 1–4	120–140	≈ 60–70%
Reinforcement stage	Skill consolidation	Weeks 5–8	140–160	≈ 70–80%
Improvement stage	Skill application and improvement	Weeks 9–12	150–170	≈ 75–85%

HR, heart rate; HRmax, estimated maximal heart rate.

Exercise load and safety were monitored in both groups during PE lessons through routine teacher observation and class management. These procedures followed the standardized PE teaching manual used for regular school physical education in China. In this study, the standardized PE teaching manual referred to school-level teaching documents developed according to official guidance for primary and secondary school PE, including syllabus requirements, semester teaching plans, lesson-plan requirements, class organization, safety management, and routine load-control principles for PE lessons. On this basis, the MTG received the study-specific mixed exercise intervention and additionally followed the staged target heart-rate ranges presented in Table 4 to guide the progressive exercise load. In contrast, the CONG did not receive any additional stage-specific heart-rate prescription but followed routine PE lessons designed according to the standardized PE teaching manual. The CONG mainly received calisthenics, general warm-up activities, basic running drills, flexibility exercises, and general physical fitness exercises commonly used in the school curriculum, without systematic cooperative game-based instruction, specialized ball-sport training, or small-sided tactical games.

The same certified PE teacher delivered the lessons for both groups to reduce teacher-related variation. The teacher had more than 3 years of teaching experience and received training on the study protocol before the intervention began. Research personnel randomly checked 10% of the lessons to verify whether the planned procedures were followed. Attendance was recorded throughout the 12-week period, and the final compliance rate was above 95%. Participants in both groups were asked not to take part in organized sport programs or systematic training outside the study. Monitoring was partly feasible because the study was conducted in a boarding school with unified daily management, regular PE scheduling, and restricted off-campus activity during school days. Activities outside the study protocol were checked through teacher logs, attendance records, monthly student self-reports, and guardian confirmation when feasible. These checks focused on whether students had joined any organized physical training or intervention-like activity outside the study.

### 2.3 Measurements and instruments

#### 2.3.1 Mental health measurement

The Mental Health Test (MHT) is a standardized screening instrument used to assess the mental health status of primary and secondary school students in China (Zhou and Toikko, 2025). The scale was revised by Zhou Bucheng and Suzuki Kiyoshi to meet the needs of mental health screening for students (Zhou, 1991), and has become a widely used mental health assessment tool in primary and secondary education (Ding et al., 2025; Zhang et al., 2025; Zheng et al., 2025).

The MHT contains 100 dichotomous items and includes eight content subscales: Learning Anxiety, Interpersonal Anxiety, Loneliness Tendency, Self-blame Tendency, Hypersensitivity Tendency, Somatic Symptoms, Fear Tendency, and Impulsivity Tendency. Higher scores indicate more severe psychological difficulties, and a score of 8 or above on any content subscale indicates an elevated risk level in that domain. The MHT also includes a 10-item validity scale for assessing response consistency; questionnaires with a validity-scale score of 7 or above were considered invalid, and no invalid questionnaires were included in the final analysis. In the current sample, internal consistency was good based on baseline responses. The Spearman-Brown split-half reliability coefficient was 0.808, and Cronbach’s α was 0.906. The MHT was chosen because it fit the short-term, school-based design of this study. Rather than measuring stable personality traits, it assesses psychological difficulty tendencies that may change with school life, peer relationships, classroom experiences, and self-evaluation. The items ask students to report recent subjective experiences and behavioral tendencies rather than knowledge or ability. Repeated testing may lead to some familiarity or response effects, but both groups completed the MHT on the same schedule. Possible retest effects were also considered through the group × time analysis. Trained researchers administered the MHT to both the MTG and CONG before and after the intervention.

#### 2.3.2 Physical fitness assessment

##### 2.3.2.1 Indicator selection and suitability for intervention

Physical fitness was assessed using test items from the National Student Physical Health Standard issued by the Ministry of Education of China. This standard is widely used in Chinese schools to monitor student fitness and provides clear testing procedures for school settings (Yang et al., 2025). For the sixth-grade students in this study, seven indicators were used: Vital Capacity (VC), 50-m Dash, 8 × 50-m Shuttle Run, Rope Skipping, Sit-ups, Sit-and-Reach, and Body Mass Index (BMI). Use of this national standard helped maintain consistency in testing across participants. The selected indicators also covered the main fitness components relevant to school PE. BMI reflects body morphological characteristics (Sweatt et al., 2024). VC and the 8 × 50-m Shuttle Run are related mainly to cardiorespiratory function and aerobic endurance (Chen et al., 2022; Ponce et al., 2023). The 50-m Dash assesses speed (Likić et al., 2024). Rope Skipping reflects coordination and rhythmic muscular endurance (Cubelo et al., 2026). Sit-ups measure trunk muscular endurance (Marković and Milošević, 2023), and Sit-and-Reach reflects flexibility (Pérez-Vigo et al., 2022). These field-based tests are also consistent with youth fitness research that uses school-friendly measures to describe health-related fitness in children (Ortega et al., 2008; Ruiz et al., 2011). In this study, the tests were used to track changes in selected school fitness components during the mixed exercise intervention. They were not intended to indicate broad clinical outcomes or overall functional capacity.

##### 2.3.2.2 Test organization and quality control

Physical fitness testing followed standardized procedures. The tests were conducted by physical education teachers who had received specific training for the assessment. These teachers did not deliver the intervention and were blinded to group allocation during testing. Pre- and post-intervention assessments were completed in the same venue, with the same equipment, at similar times of day, and under comparable weather conditions. Each testing session began with a group warm-up, and students were given adequate rest between test items to reduce fatigue-related influence on performance. Because these indicators were part of the routine national school physical fitness assessment system, the sixth-grade participants had previously completed the same standardized test items in Grade 5, which reduced the likelihood that post-test changes were mainly attributable to first-time exposure to the testing procedures. To reduce measurement error, the same timing methods and counting criteria were used for all timing/counting items, and the results were recorded and verified on site (Table 5).

**TABLE 5:** Physical fitness test items and score recording methods.

Test indicator	Unit	Trials	Recording method
BMI	kg/m^2^	–	Calculated from height and weight
Vital capacity	mL	2	Maximum value recorded
50-m dash	s	2	Best time recorded
8 × 50-m shuttle run	s	1	Completion time
Rope skipping	Times/min	2	Best score recorded
Sit-ups	Times/min	2	Best score recorded
Sit-and-reach	cm	2	Best distance recorded

BMI, body mass index. The dash indicates that no performance trial was conducted because BMI was calculated from height and weight.

#### 2.3.3 Study particularities and safety monitoring

This study was reported in accordance with the Consolidated Standards of Reporting Trials (CONSORT) 2025 guidelines. The trial was retrospectively registered at ClinicalTrials.gov (Identifier: NCT07452302; registered on 04 March 2026; publicly accessible registry).^1^ The research protocol followed the Declaration of Helsinki and was approved by the Ethics Committee of Anhui Normal University (Approval No. AHNU-EJ2025069). Written informed consent was obtained from the legal guardians of all participants, and written assent was obtained from all children prior to enrollment. No interim analyses were planned or conducted, and no stopping guidelines were established, as this study was a low-risk behavioral intervention with a predefined 12-week duration. All 40 randomized participants completed the study and were included in the final analysis according to their original group assignment. No participants withdrew, no outcome data were missing, and no data imputation was required. No ancillary analyses, including subgroup or sensitivity analyses, were performed beyond the prespecified protocol. Adverse events, including exercise-related injuries, fainting, or severe physical discomfort, were monitored throughout the 12-week intervention period via daily teacher logs and student self-reports. No adverse events, injuries, or other harms were identified or reported in either group during the study period.

### 2.4 Statistical analysis

Data processing was conducted using GraphPad Prism 10.0 (GraphPad Software, La Jolla, CA, United States). Descriptive statistics for continuous data are presented as means ± standard deviations (Mean ± SD). Normality of the data for psychological and physical variables was assessed using the Shapiro–Wilk test to ensure suitability for parametric testing. At baseline, the comparability of MHT scores and fitness indicators between the groups was assessed using independent-samples *t-*tests.

To assess intervention-related changes, a 2 (time: before intervention vs. after intervention) × 2 (group: CONG vs. MTG) repeated-measures ANOVA was used. Mental health and physical fitness outcomes were used as dependent variables. This analysis assessed the main effects of group and time, as well as the key group × time interaction. Given that the time factor had only two levels, the assumption of sphericity was inherently met. Intervention-related change was interpreted primarily according to the group × time interaction rather than within-group pre–post change alone, which provided a basis for distinguishing MTG-associated changes from general temporal or repeated-measurement effects that could occur in both groups.

To quantify the magnitude of the effects, two types of effect sizes were calculated:

Partial eta-squared (*η*^2^*_*p*_*) was calculated to quantify the proportion of variance attributable to each ANOVA effect. Effect sizes were derived directly from the ANOVA output using standard procedures implemented in the statistical software.

Cohen’s *d*: For interaction effects, a standardized difference-in-differences (ΔΔ) method was used. Cohen’s d was calculated by dividing the net change difference (ΔMTG–ΔCONG) by the pooled baseline standard deviation (SD_pooled_baseline). This method was chosen to characterize the degree of variability of the intervention effect relative to the initial population.

If a significant interaction was found, a *post-hoc* simple effects analysis was performed using the Bonferroni correction method. The precision of the results was indicated by reporting 95% confidence intervals (95% CI) for the mean differences of the interaction term. Statistical significance was defined as *p* < 0.05 (two-tailed) for all tests.

## 3 Results

Data from all 40 randomized participants (MTG: *n* = 20; CONG: *n* = 20) were available for analysis. Means, standard deviations (SD), effect sizes, and 95% confidence intervals (CI) are presented in the following tables. To improve transparency in this small-sample intervention study, participant-level pre–post trajectories and individual change scores are also presented in the figures.

### 3.1 Baseline comparison of physical fitness and mental health indicators between the MTG and CONG

Baseline comparability between the MTG and the CONG was assessed using independent-samples *t-*tests for both physical fitness metrics and mental health indicators (Tables 6, 7).

**TABLE 6:** Baseline comparison of physical fitness indicators between the MTG and CONG.

Indicator	CONG (*n* = 20)	MTG (*n* = 20)	*t*	*p*
Rope skipping, times/min	117.10 ± 14.88	116.70 ± 10.90	−0.09	0.925
Sit-ups, times/min	24.50 ± 5.76	24.20 ± 6.37	0.15	0.880
50-m dash, s	10.13 ± 0.88	10.37 ± 0.72	−0.90	0.374
BMI, kg/m^2^	18.12 ± 0.68	18.38 ± 0.90	1.10	0.279
Vital capacity, mL	2315.45 ± 190.41	2314.05 ± 211.89	0.02	0.983
8 × 50-m shuttle run, s	129.80 ± 7.69	131.95 ± 9.06	−0.79	0.435
Sit-and-reach, cm	6.25 ± 4.11	6.63 ± 4.26	0.15	0.880

Values are presented as mean ± SD. CONG, control group; MTG, mixed training group; BMI, body mass index. *t* and *p*-values were derived from independent-samples *t*-tests.

**TABLE 7:** Baseline comparison of psychological health indicators between the MTG and CONG.

Indicator	CONG (*n* = 20)	MTG (*n* = 20)	*t*	*P*
Learning anxiety	9.10 ± 0.89	9.00 ± 1.73	0.224	0.824
Interpersonal anxiety	5.55 ± 1.56	5.60 ± 2.13	−0.089	0.930
Loneliness tendency	5.95 ± 0.97	5.70 ± 1.65	0.593	0.557
Self-blame tendency	8.00 ± 1.05	8.05 ± 1.28	−0.138	0.891
Hypersensitivity tendency	8.15 ± 0.85	8.25 ± 1.44	−0.279	0.782
Somatic symptoms	8.54 ± 1.24	8.40 ± 2.27	0.255	0.800
Fear tendency	4.90 ± 1.04	4.40 ± 2.46	0.858	0.396
Impulsivity tendency	7.45 ± 1.07	7.50 ± 1.91	−0.113	0.911
MHT total score	57.65 ± 4.28	56.90 ± 7.43	−0.391	0.698

Values are presented as mean ± SD. MHT, Mental Health Test. Higher scores indicate greater psychological difficulties. *t* and *p*-values were derived from independent-samples *t*-tests.

In terms of physical fitness, no significant differences were found in performance measures for Rope Skipping, Sit-ups, 50-m Dash, 8 × 50-m Shuttle Run, Vital Capacity, Sit-and-Reach, or BMI (all *p* > 0.05). This suggests that the MTG and CONG had comparable physical fitness profiles before the intervention.

Similarly, for mental health, the analysis revealed no significant differences between the groups in scores for Learning Anxiety, Interpersonal Anxiety, Loneliness Tendency, Self-Blame Tendency, Hypersensitivity Tendency, Somatic Symptoms, Fear Tendency, Impulsivity Tendency, and MHT total score (*p* > 0.05). These results suggest that the groups were well matched regarding psychological status at the study outset.

### 3.2 Effects of the mixed intervention of sports games and ball sports on mental health indicators

To examine changes in psychological health following the combined sports-game and ball-sport intervention, MHT total and subscale scores were analyzed using a 2 × 2 repeated-measures ANOVA (Table 8).

**TABLE 8:** Changes in psychological health indicators before and after the 12-week intervention.

Indicator	CONG pre	CONG post	MTG pre	MTG post	Time *p*(*η* ^2^*p*)	Group *p*(*η* ^2^*p*)	Interaction *p*(*η* ^2^*p*)	ΔΔ [95% CI]	*d*
Primary outcome									
MHT total score	57.65 ± 4.28	55.05 ± 2.04	56.90 ± 7.43	47.80 ± 4.21*#	< 0.001 (0.523)	0.003 (0.212)	< 0.001(0.253)	−6.50[−10.17, −2.83]	−1.07
Secondary outcomes									
Self-blame tendency	8.0 ± 1.1	7.9 ± 0.9	8.1 ± 1.3	6.2 ± 0.9*#	< 0.001(0.288)	0.001(0.256)	0.002(0.226)	−1.70[−2.73, −0.67]	−1.41
Interpersonal anxiety	5.6 ± 1.6	5.8 ± 1.4	5.6 ± 2.2	4.4 ± 1.6*#	0.047(0.100)	0.196(0.044)	0.007(0.178)	−1.40[−2.39, −0.41]	−0.73
Loneliness tendency	6.0 ± 1.0	5.7 ± 0.9	5.7 ± 1.7	4.4 ± 1.2*#	0.005(0.191)	0.011(0.157)	0.069(0.084)	−1.00[−2.08, 0.08]	−0.72
Impulsivity tendency	7.5 ± 1.1	7.2 ± 0.9	7.5 ± 2.0	6.3 ± 1.3*#	0.005(0.194)	0.235(0.037)	0.072(0.083)	−0.95[−1.99, 0.09]	−0.59
Learning anxiety	9.1 ± 0.9	8.1 ± 0.9*	9.0 ± 1.8	7.0 ± 1.7*#	< 0.001 (0.439)	0.094 (0.072)	0.098(0.070)	−1.00 [−2.08, 0.18]	−0.70
Somatic symptoms	8.6 ± 1.3	8.2 ± 0.8	8.4 ± 2.3	7.4 ± 0.9*	0.017(0.141)	0.192(0.044)	0.269(0.032)	−0.65[−1.82, 0.52]	−0.35
Fear tendency	4.9 ± 1.1	4.7 ± 0.8	4.4 ± 2.5	4.3 ± 1.9	0.601(0.007)	0.328(0.025)	0.823(0.001)	0.15[−1.19, 1.49]	0.08
Hyper-sensitivity tendency	8.2 ± 0.9	7.8 ± 0.6	8.3 ± 1.5	7.9 ± 0.9	0.109(0.066)	0.683(0.004)	> 0.999(0.000)	0.00 [−0.86, 0.86]	0.00

Values are presented as mean ± SD. Primary outcomes are presented first, followed by secondary outcomes. CONG, control group; MTG, mixed training group; MHT, Mental Health Test; η^2^*p*, partial eta squared; ΔΔ = (MTGpost − MTGpre) − (CONGpost − CONGpre). Cohen’s d represents the standardized difference-in-differences effect size.

*p < 0.05 compared with baseline (Pre).

#*p* < 0.05 compared with CONG at posttest.

At the overall level, MHT total scores in the MTG decreased significantly after the intervention compared with baseline (56.90 ± 7.43 vs. 47.80 ± 4.21, *p* < 0.05) and were significantly lower than those of the CONG at post-test (47.80 ± 4.21 vs. 55.05 ± 2.04, *p* < 0.05). Significant main effects of Time (*p* < 0.001, *η*^2^*_*p*_* = 0.523) and Group (*p* = 0.003, *η*^2^*_*p*_* = 0.212) were observed, along with a significant Time × Group interaction (*p* < 0.001, *η*^2^*_*p*_* = 0.253). The standardized net effect for the MHT total score favored the MTG (*d* = −1.07, ΔΔ = −6.50, 95% CI: −10.17 to −2.83).

For the MHT subscales, significant Time × Group interactions were observed for Interpersonal Anxiety (*p* = 0.007, *η*^2^*_*p*_* = 0.178, *d* = −0.73) and Self-Blame Tendency (*p* = 0.002, *η*^2^*_*p*_* = 0.226, *d* = −1.41), indicating greater reductions in the MTG than in the CONG. Loneliness Tendency showed a significant main effect of Group (*p* = 0.011, *η*^2^*_*p*_* = 0.157), but the Time × Group interaction did not reach statistical significance (*p* = 0.069, *η*^2^*_*p*_* = 0.084, *d* = −0.72). Learning Anxiety and Impulsivity Tendency showed significant Time effects (both *p* < 0.01), but their Time × Group interactions were not statistically significant (*p* = 0.098, *η*^2^*_*p*_* = 0.070, *d* = −0.70; and *p* = 0.072, *η*^2^*_*p*_* = 0.083, *d* = −0.59, respectively). Therefore, these non-significant interaction effects were not interpreted as evidence of intervention-specific effects.

No significant Time × Group interactions were detected for Hypersensitivity Tendency (*η*^2^*_*p*_* ≈ 0), Fear Tendency (*η*^2^*_*p*_* = 0.001), or Somatic Symptoms (*p* = 0.269, *η*^2^*_*p*_* = 0.032). Somatic Symptoms showed a significant main effect of Time (*p* = 0.017, *η*^2^*_*p*_* = 0.141), suggesting that the observed change was not specific to the intervention.

### 3.3 Effects of the mixed intervention of sports games and ball sports on physical fitness indicators

To examine physical fitness changes following the combined sports-game and ball-sport intervention, a 2 × 2 repeated-measures ANOVA was conducted (Table 9).

**TABLE 9:** Changes in physical fitness indicators before and after the 12-week intervention.

Indicator	CONG pre	CONG post	MTG pre	MTG post	Time *p*(*η* ^2^*p*)	Group *p*(*η* ^2^*p*)	Interaction *p*(*η* ^2^*p*)	ΔΔ [95% CI]	d
Primary outcomes									
Vital capacity, mL	2315.5 ± 195.4	2296.5 ± 197.4	2314.1 ± 217.4	2496.0 ± 215.2*#	< 0.001(0.606)	0.132(0.059)	< 0.001(0.700)	201.40[158.10, 244.70]	0.97
8 × 50-m shuttle run, s	129.8 ± 7.9	129.3 ±8.0	132.0 ± 9.3	123.7 ± 8.2*#	< 0.001(0.801)	0.515(0.011)	< 0.001(0.755)	−7.75[−9.20, −6.30]	−0.90
Secondary outcomes									
50-m dash, s	10.1 ± 0.9	10.0 ± 0.7	10.4 ± 0.7	9.6 ± 1.0*	< 0.001(0.378)	0.752(0.003)	0.002(0.223)	−0.63[−1.01, −0.24]	−0.78
Rope skipping, times/min	117.1 ± 15.3	117.8 ± 11.5	116.7 ± 11.1	127.1 ± 7.8*#	0.011(0.158)	0.158(0.052)	0.024(0.127)	9.70[1.35, 18.05]	0.73
Sit-and-reach, cm	6.3 ± 4.2	6.0 ± 4.4	6.6 ± 4.4	7.2 ± 4.9*	0.375(0.021)	0.571(0.009)	0.040(0.106)	0.83[0.04, 1.62]	0.19
BMI, kg/m^2^	18.1 ± 0.7	18.2 ± 0.6	18.4 ± 0.9	18.3 ± 1.0	0.838(0.001)	0.414(0.018)	0.225(0.039)	−0.12[−0.32, 0.08]	−0.15
Sit-ups, times/min	24.5 ± 5.9	24.7 ± 5.2	24.2 ± 6.5	25.2 ± 5.7	0.534(0.010)	0.950(0.000)	0.678(0.005)	0.80[−3.07, 4.67]	0.13

Values are presented as mean ± SD. Primary outcomes are presented first, followed by secondary outcomes. CONG, control group; MTG, mixed training group; BMI, body mass index; η^2^*p*, partial eta squared; ΔΔ = (MTGpost − MTGpre) − (CONGpost − CONGpre). Cohen’s d represents the standardized difference-in-differences effect size.

*p < 0.05 compared with baseline (Pre).

#*p* < 0.05 compared with CONG at posttest.

Regarding physical fitness, the MTG showed significant improvements in several fitness indicators after the intervention and showed better post-intervention performance than the CONG on selected indicators, suggesting selective improvements in physical fitness. Specifically, the MTG showed significant improvements in Vital Capacity (Pre 2314.1 ± 217.4, Post 2496.0 ± 215.2; CONG Post 2296.5 ± 197.4), 50-m Dash (Pre 10.4 ± 0.7, Post 9.6 ± 1.0; CONG Post 10.0 ± 0.7), 8 × 50-m Shuttle Run (Pre 132.0 ± 9.3, Post 123.7 ± 8.2; CONG Post 129.3 ± 8.0), Rope Skipping (Pre 116.7 ± 11.1, Post 127.1 ± 7.8; CONG Post 117.8 ± 11.5), and Sit-and-Reach (Pre 6.6 ± 4.4, Post 7.2 ± 4.9; CONG Post 6.0 ± 4.4). All these indicators showed significant within-group improvements for the MTG and were significantly better than the CONG at post-test (all *p* < 0.05).

Further analysis using repeated-measures ANOVA revealed significant Time × Group interaction effects for Vital Capacity (*p* < 0.001, *η*^2^*_*p*_* = 0.700, *d* = 0.97, ΔΔ = 201.40, 95% CI: 158.10–244.70), 50-m Dash (*p* = 0.002, *η*^2^*_*p*_* = 0.223, *d* = −0.78, ΔΔ = −0.63, 95% CI: −1.01 to −0.24), and 8 × 50-m Shuttle Run (*p* < 0.001, *η*^2^*_*p*_* = 0.755, *d* = −0.90, ΔΔ = −7.75, 95% CI: −9.20 to −6.30). These results showed greater pre–post improvements in the MTG than in the CONG, particularly in aerobic capacity and speed-agility. The Rope Skipping test also showed a significant interaction effect (*p* = 0.024, *η*^2^*_*p*_* = 0.127, *d* = 0.73, ΔΔ = 9.70, 95% CI: 1.35–18.05). Although the Sit-and-Reach test displayed a significant interaction (*p* = 0.040, *η*^2^*_*p*_* = 0.106), the magnitude of standardized improvement was relatively smaller (*d* = 0.19, ΔΔ = 0.83, 95% CI: 0.04–1.62).

For the remaining indicators, no significant Time × Group interactions were found in BMI (*p* = 0.225, *η*^2^*_*p*_* = 0.039, *d* = −0.15) or Sit-ups (*p* = 0.678, *η*^2^*_*p*_* = 0.005, *d* = 0.13), with no significant within-group or between-group differences observed (all *p* > 0.05).

**FIGURE 3:**
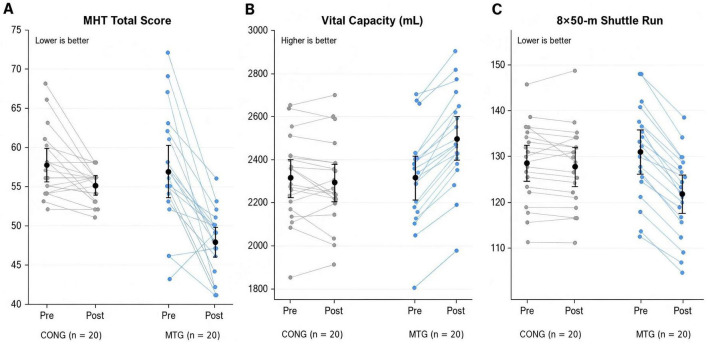
Participant-level pre–post trajectories for primary outcomes. **(A)** MHT total score; **(B)** vital capacity; **(C)** 8 × 50-m shuttle run. Individual trajectories are shown for each participant in the control group (CONG) and mixed training group (MTG). Each point represents one participant, and connecting lines indicate within-participant pre–post changes. Black dots and error bars represent group means and 95% confidence intervals. Lower values indicate improvement for MHT total score and 8 × 50-m shuttle run, whereas higher values indicate improvement for vital capacity.

**FIGURE 4:**
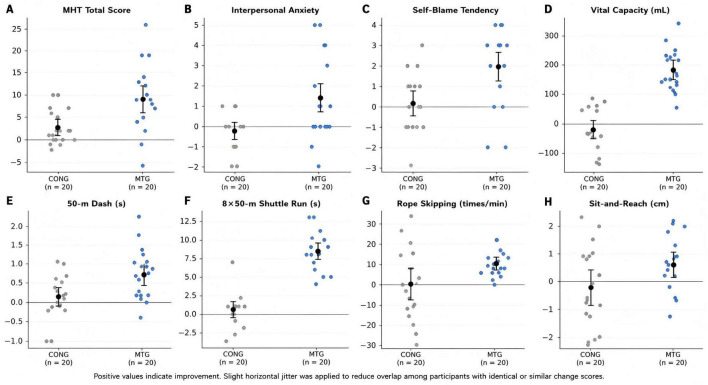
Participant-level change scores for primary and selected intervention-related outcomes. **(A)** MHT total score; **(B)** interpersonal anxiety; **(C)** self-blame tendency; **(D)** vital capacity; **(E)** 50-m dash; **(F)** 8 × 50-m shuttle run; **(G)** rope skipping; **(H)** sit-and-reach. Each point represents one participant. Black dots and error bars represent group mean changes and 95% confidence intervals. For all panels, positive values indicate improvement. Change scores were calculated as pre minus post for outcomes where lower values indicate better performance or fewer psychological difficulties, and as post minus pre for outcomes where higher values indicate better performance. Slight horizontal jitter was applied to reduce overlap among participants with identical or similar change scores. CONG, control group; MTG, mixed training group.

To further examine individual-level outcome changes, participant-level visualizations are presented in Figures 3, 4. Figure 3 shows individual pre–post trajectories for the three prespecified primary outcomes. For MHT total score, most participants in the MTG showed a downward trajectory from pre- to post-intervention, whereas changes in the CONG were smaller and less consistent. For vital capacity, most MTG participants showed upward trajectories, while the CONG showed limited overall change. For the 8 × 50-m shuttle run, most MTG participants showed shorter completion times after the intervention, whereas the CONG showed relatively small and heterogeneous changes.

Figure 4 presents individual change scores for the primary and selected intervention-related outcomes. Positive values indicate improvement across all panels. The MTG showed a generally more favorable distribution of individual change scores for MHT total score, interpersonal anxiety, self-blame tendency, vital capacity, 50-m dash, 8 × 50-m shuttle run, rope skipping, and sit-and-reach. These participant-level patterns were consistent with the significant Time × Group interactions reported in Tables 8, 9, while also showing the degree of individual variability within each group.

Overall, this mixed intervention was associated with selective improvements in key psychological and physical indicators of LBC within limited classroom time, rather than comprehensive or generalized improvement. In terms of mental health, statistically significant intervention-related effects were mainly reflected in MHT total score, interpersonal anxiety, and self-blame tendency. Learning anxiety, loneliness tendency, and impulsivity tendency showed some within-group or main-effect changes, but their Time × Group interactions did not reach statistical significance and were therefore not interpreted as intervention-specific effects. In terms of physical fitness, the MTG showed greater intervention-related changes in vital capacity, 50-m dash, 8 × 50-m shuttle run, rope skipping, and sit-and-reach, mainly involving cardiorespiratory function, aerobic endurance, speed, coordination, and flexibility. In contrast, no significant intervention-related changes were observed in BMI or sit-ups. The participant-level figures further indicated that these findings were not solely dependent on group mean differences, but were accompanied by generally more favorable individual-level changes in the MTG for the primary and selected secondary outcomes.

## 4 Discussion

Overall, this 12-week randomized controlled trial showed a selective pattern of short-term changes in psychological and physical fitness outcomes among LBC. The mixed PE intervention was not associated with broad improvements across all measured indicators. Compared with the CONG, the MTG showed more favorable changes in MHT total score, Interpersonal Anxiety, Self-Blame Tendency, Vital Capacity, 50-m Dash, 8 × 50-m Shuttle Run, Rope Skipping, and Sit-and-Reach. By contrast, Hypersensitivity Tendency, Fear Tendency, Somatic Symptoms, BMI, and Sit-ups showed no clear intervention-specific changes. Learning Anxiety, Loneliness Tendency, and Impulsivity Tendency showed within-group or main-effect changes, but their Time × Group interactions were not statistically significant; these outcomes were therefore interpreted as time-related or non-specific changes rather than intervention-specific effects. The results point to a selective pattern of change: improvements were mainly found in certain psychological difficulty tendencies and school-relevant fitness components, rather than across the full range of physical and mental health outcomes in LBC.

For psychological outcomes, significant Time × Group interactions were found for MHT total score, Interpersonal Anxiety, and Self-Blame Tendency. In practical terms, children in the MTG showed greater reductions in these psychological difficulty tendencies than those in the CONG after the 12-week intervention. These effects were not evenly distributed across all psychological dimensions. They were mainly concentrated in domains related to social interaction, peer evaluation, and self-related emotional responses. Learning Anxiety, Loneliness Tendency, and Impulsivity Tendency showed within-group or main-effect changes, but their Time × Group interactions were not statistically significant. These outcomes were therefore treated as time-related or non-specific changes rather than clear intervention-specific effects.

This pattern can be understood in relation to the intervention design and the theoretical view that children’s psychological adjustment is shaped by repeated social experiences within everyday school contexts. Interpersonal Anxiety and Self-Blame Tendency are closely related to peer relationships, social evaluation, failure experiences, and self-related emotional responses (Chiu et al., 2021; Higgins, 1987; Watson and Friend, 1969). Sports games may provide safer opportunities for group participation through rule-based involvement, role taking, cooperative tasks, and low-exclusion activity settings, which are theoretically linked to relatedness and perceived competence in classroom participation (O’Connor et al., 2024; Xing et al., 2025). Ball-sport activities, through passing, receiving, positioning, offensive and defensive transitions, and shared team goals, may further increase opportunities for peer interaction in PE classes (Ali, 2024; Shi, 2024). When these two activity forms are combined, classroom evaluation may become less centered on individual performance, and failure-related pressure may be partly distributed through team tasks, shared goals, and peer support. This may help explain why the psychological changes were more evident in Interpersonal Anxiety and Self-Blame Tendency, which are sensitive to peer evaluation and failure-related experiences.

This finding is broadly consistent with previous evidence on physical activity and youth mental health. Biddle and Asare’s review showed that physical activity is related to depression, anxiety, self-esteem, and cognitive functioning in young people (Biddle and Asare, 2011). Recchia et al. (2023) meta-analysis, which included 21 trials, reported a small but significant reduction in depressive symptoms after physical activity interventions in children and adolescents (*g* = −0.29). Compared with these studies, the present findings did not show broad improvement across all psychological dimensions. Instead, the effects were more concentrated in social-emotional dimensions. This suggests that physical activity may not improve all psychological difficulties in the same way or to the same degree. Its effects may depend on the source of the psychological difficulty, the structure of classroom participation, and the match between the intervention duration and the target psychological dimension.

From a psychological perspective, children’s mental health development depends on stable emotional support, a sense of security, positive peer relationships, reasonable self-evaluation, and the gradual development of behavioral regulation (Bowlby, 1969; Rubin et al., 2006). For LBC, long-term parent-child separation, insufficient emotional companionship, academic pressure, and unstable peer relationships may continue to shape their sense of belonging, self-worth, and emotion regulation. The lack of significant Time × Group interactions for Learning Anxiety, Loneliness Tendency, and Impulsivity Tendency needs to be read carefully. These results do not negate the psychological value of physical activity for LBC. A more likely explanation is that short-term changes within PE lessons may first affect social-emotional experiences closely tied to classroom interaction. By contrast, learning anxiety, loneliness, and impulsive tendencies may be shaped by longer-term family, academic, and peer experiences, and may require more sustained support. In this sense, physical activity should not be viewed only as a short-term intervention for LBC. It may also function as part of everyday school life, where peer cooperation, teacher support, rule-based participation, and emotional expression are repeatedly practiced.

Differences in psychological responses may also reflect sample size, baseline psychological status, classroom engagement, prior peer relationships, and the novelty of the intervention. Randomization and Time × Group interactions helped distinguish intervention-related changes from general time-related changes, but several process variables were not directly measured, including peer acceptance, sense of belonging, self-evaluation, and attribution style. For this reason, the proposed psychological explanations remain tentative. From a school-based perspective, the observed reductions may still reflect meaningful improvement in psychological difficulty tendencies, but they should not be taken as evidence of clinical recovery because the MHT is a screening rather than diagnostic instrument. The relatively large psychological effect sizes should also be viewed as preliminary and sample-specific estimates given the small sample size. These findings align with previous evidence that physical activity may support youth mental health, while also indicating that psychological responses depend on the source of the difficulty, the form of classroom participation, and the continuity of the intervention.

For physical fitness outcomes, the findings provide partial support for the primary hypothesis. The MTG showed more favorable intervention-related changes in Vital Capacity, 50-m Dash, 8 × 50-m Shuttle Run, Rope Skipping, and Sit-and-Reach. BMI and Sit-ups did not show clear intervention-specific changes. This pattern suggests that the mixed PE program mainly affected fitness components closely linked to the activities practiced in class and to the planned exercise intensity. It did not operate as a general physical training program for all fitness domains.

The practical significance of these results should be considered in the context of school PE. Cardiorespiratory fitness, speed-agility, endurance, coordination, and flexibility are often used in youth fitness research and school assessment to describe health-related fitness and PE-relevant performance (Ortega et al., 2008; Ruiz et al., 2011). The improvements in Vital Capacity, 50-m Dash, 8 × 50-m Shuttle Run, and Rope Skipping therefore suggest better performance in several fitness components that are directly relevant to PE participation and school physical activity. At the same time, the interpretation should remain bounded. These results should not be interpreted as evidence of broad clinical or functional improvement. The Sit-and-Reach result was also modest in practical terms, even though it reached statistical significance, because the observed change was small (ΔΔ = 0.83, *d* = 0.19).

The pattern of fitness change was closely related to the intervention content. Across the 12 weeks, cooperative games, ball-sport practice, and staged load control repeatedly involved running, short sprints, changes of direction, coordinated movement, and peer-based participation. These activities were more likely to affect fitness components used directly in PE lessons. Basketball- and soccer-based activities require students to run, sprint, pass, receive, shift between offensive and defensive situations, and change direction quickly (Spoelstra, 2025). Such movement demands are relevant to cardiorespiratory function, aerobic endurance, and speed-agility in ordinary PE classes. The sports games also included rhythmic, coordinated, and task-oriented movements (Wahyuniati et al., 2025). Because these games were organized in a relatively low-pressure format, students may have been more willing to engage in coordination-related movement tasks. This link between activity content and measured outcomes helps explain why the clearest improvements appeared in cardiorespiratory function, endurance, speed, and coordination-related measures.

The unchanged BMI and Sit-up performance also fit the intervention design. The program did not include weight-control strategies or targeted strength-endurance training. Changes in body composition usually require longer-term regulation of energy balance, whereas strength-related adaptations depend more on specific loading stimuli (Ihsan et al., 2025; Nuzzo, 2023). Previous research has also shown that body composition and muscular strength are more likely to change when interventions include sustained energy-expenditure control or systematically prescribed strength loads (Li et al., 2022; Staśkiewicz-Bartecka et al., 2024; Williams et al., 2025). These outcomes may be less responsive to short-term, non-targeted classroom activities. The relative stability of BMI and strength-endurance indicators in this study is consistent with these physiological adaptation characteristics.

Several statistically significant findings still require caution. The Time × Group interactions for Vital Capacity and the 8 × 50-m Shuttle Run were significant, but the corresponding partial eta-squared values were large. Because partial eta-squared is sensitive to design-specific error variance, it may be inflated in small repeated-measures samples, especially when within-participant changes are relatively consistent. These effect sizes were interpreted together with raw change estimates, standardized net effects, and 95% confidence intervals. They should be viewed as sample-specific estimates of intervention-related change rather than definitive evidence of unusually large population-level effects. In addition, these fitness indicators were standardized school-based tests that participants had previously completed in routine national school fitness assessments, which likely reduced first-exposure effects. Even so, repeated-testing or task-familiarity effects cannot be completely excluded. For this reason, the interpretation of intervention-related changes was based mainly on Time × Group interactions rather than within-group pre–post changes alone.

The value of the mixed PE model may lie not only in the observed outcomes, but also in its fit with the real conditions of routine school PE. Previous studies have suggested that sports games, ball-sport activities, and school-based physical activity can support children’s psychological and physical development (Bailey, 2006; Eime et al., 2013; Mo et al., 2024). The present study extends this evidence by testing an approach embedded in ordinary PE lessons, without adding extracurricular training time, changing school facilities, or requiring complex equipment. Rather than introducing a separate training system, the intervention adjusted modifiable elements of routine PE, including content structure, activity organization, participation format, and staged exercise-load control. In this sense, targeted reorganization of regular PE may offer a practical school-based pathway for combining physical load with meaningful peer interaction in ways that may support selected psychological difficulty tendencies and PE-relevant fitness components among LBC.

Although the present trial focused specifically on rural left-behind children, the mixed PE model may also have implications for age-matched children without left-behind experience. The intervention was implemented within regular PE lessons and relied on common school activities, cooperative games, ball-sport tasks, and staged load control rather than specialized therapeutic procedures. Therefore, benefits related to peer interaction, participation motivation, motor-skill practice, and school-relevant fitness may also be relevant to broader sixth-grade student populations. However, because children without left-behind experience were not included in this trial, such applicability should be regarded as a pedagogical implication rather than an empirically confirmed effect. Future studies should compare left-behind and non-left-behind children to determine whether the same PE structure produces similar or differential benefits across child groups.

This model was not a simple addition of sports games and ball sports, but an attempt to use their complementary strengths. Sports games provide low-threshold, cooperative, and relatively low-pressure participation settings, which may help children enter group activities more easily. Ball-sport activities require more movement skills, continuous running, and team coordination, and may provide more stable physical-conditioning stimuli and peer-cooperation contexts. Their combination may compensate for the limited exercise load of game-only activities while reducing the participation pressure and competitive burden that may occur in ball-sport-only training. The contribution of this study lies in integrating psychological participation and physical-conditioning stimuli within routine PE, with preliminary evidence of selective improvements in some psychological difficulty tendencies and school-relevant fitness components.

This interpretation still requires caution. The study tested an integrated model composed of cooperative sports games, basketball- and soccer-based activities, and staged exercise-load control, not the independent effect of any single component. The current design cannot determine how much of the observed change was attributable to sports games, ball-sport tasks, or exercise-load progression separately. The greater structure and participation-oriented format of the MTG were intentional pedagogical features, but possible non-specific attention, novelty, or Hawthorne-type effects cannot be fully excluded because no attention-matched or novelty-matched control condition was included. The findings should be interpreted within the present sample, intervention period, and school context. Their broader applicability requires further examination, as discussed in the following limitations and future directions section.

## 5 Limitations and future directions

Several limitations should be acknowledged. First, the sample size was relatively small, and all participants were recruited from a single rural boarding primary school, which may limit generalizability. School context, peer influence within the same campus, and possible clustering at the school level were not fully controlled. The boarding-school setting made it easier to monitor organized activities, but compliance was still assessed mainly through teacher logs, attendance records, and student self-reports rather than objective physical activity monitoring. Some spontaneous or unrecorded activity outside the intervention may have been missed. The study also lasted only 12 weeks and did not include follow-up testing, so the persistence of the observed psychological and physical changes remains unknown. Another limitation is that the intervention combined cooperative games, basketball- and soccer-based activities, and staged exercise-load control in one program. Without separate intervention arms, the contribution of each component cannot be isolated. The focus on LBC also means that the findings should not be directly extended to other child groups or developmental stages. Future research should include larger multi-site samples, longer follow-up periods, objective activity monitoring where feasible, and dismantling or factorial designs to examine the durability, generalizability, and component-specific effects of this mixed PE model.

## 6 Conclusion

Results from this 12-week randomized controlled trial indicate that the integration of cooperative games and ball sports into regular PE classes was associated with selective improvements in psychosocial health and physical fitness among rural LBC. The intervention mainly affected social-emotional psychological difficulty tendencies and fitness components linked to cardiorespiratory function, aerobic endurance, speed, and coordination. These changes were observed within regular PE classes and did not require additional extracurricular exercise time. The findings provide preliminary evidence that adjusting the structure of existing PE lessons may support specific developmental outcomes in this group. Given the modest sample size, single-site design, and absence of follow-up assessment, the results should be interpreted with caution. Larger multi-site studies with longer follow-up are needed to confirm these findings and to examine whether this mixed exercise model can be applied in broader school contexts.

## Data Availability

The original contributions presented in the study are included in the article/Supplementary material, further inquiries can be directed to the corresponding author.

## Footnotes

1
https://clinicaltrials.gov/study/NCT07452302
